# Immunotherapy for head and neck squamous cell carcinoma

**DOI:** 10.1007/s12254-016-0270-8

**Published:** 2016-06-20

**Authors:** Thorsten Fuereder

**Affiliations:** Dept. of Internal Medicine I & CCC, Medical University of Vienna, Währinger Gürtel 18–20, 1090 Vienna, Austria

**Keywords:** head and neck squamous cell carcinoma, immunotherapy, checkpoint inhibitor, PDL-1, PD-1

## Abstract

Over the past years immuno-oncology has evolved and become a novel promising strategy for cancer therapy. Immune checkpoint inhibitors such as pembrolizumab or nivolumab, which target the interaction between programmed death receptor 1/programmed death ligand 1 (PD-1/PDL-1) and PDL-2, have been recently approved for the treatment of various malignancies and are currently being investigated in clinical phase III trials for head and neck squamous cell carcinoma (HNSCC). Data available from these trials indicate substantial activity accompanied by a favorable safety and toxicity profile in this patient population. This review article focuses on the molecular background, gives an overview of current clinical data of checkpoint inhibitors in HNSCC, and points out future challenges such as the need for appropriate biomarkers for these novel compounds.

## Introduction

**Fig. 1:**
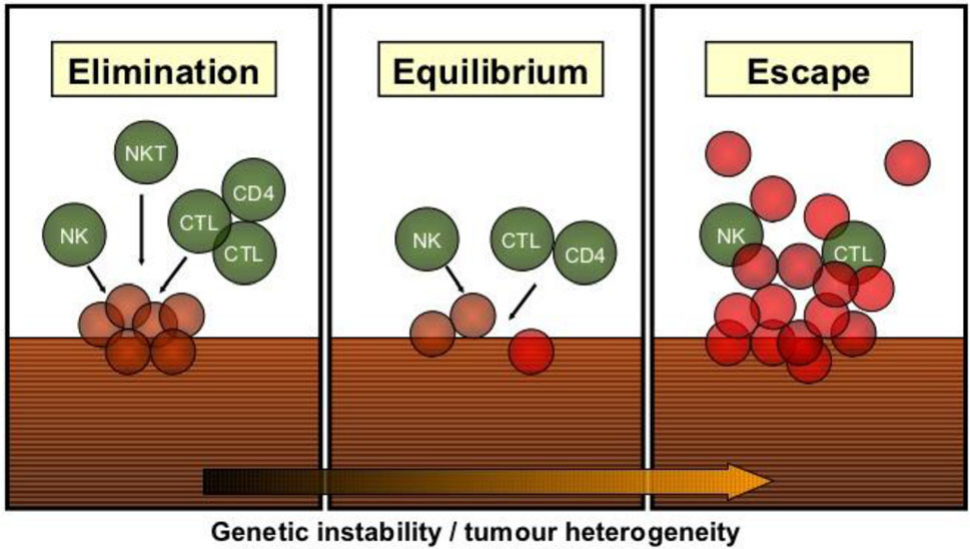
The principle of immunoediting consisting of tumor elimination, an equilibrium phase, and tumor escape

Beginning from the late 19th century, when William Cloey treated cancer with a mixture of killed bacteria, until the modern era of checkpoint inhibitors immunotherapy has evolved to a powerful weapon for anticancer treatment. The relevance of an intact host immune response for cancer prevention has been initially demonstrated in animal experiments, which showed an increased incidence of tumors in mice with deficiencies in the innate or adaptive immune system [1, 2]. Consistently, persons with HIV/AIDS have a 2–6 fold increased risk for the development of oropharyngeal cancer compared to the general population [3]. It is generally accepted that immunosurveillance, i. e., the recognition and elimination of malignant cells by the immune system constantly occurs in humans as well. Based on the concept of immunosurveillance the term immunoediting was coined. Immunoediting is a dynamic process consisting of tumor elimination, equilibrium, and tumor escape (Fig. 1; [4]). Tumor elimination represents the successful eradication of the evolving tumor by the immune system. However, if the tumor is not completely destroyed, tumor cells might enter an equilibrium state, where the immune system controls tumor outgrowth but elimination remains incomplete [2, 5]. The equilibrium might last for years but negatively selects tumor cells, which can evade the immune system.

## Relevance of immune cell infiltration in head and neck cancer

Head and neck squamous cell carcinoma (HNSCC) induces an immune suppressive state via various mechanisms. Patients with HNSCC have altered lymphocyte homeostasis (mainly reduced levels of CD3+, CD4+, and CD8+ T cells) compared to healthy controls [6]. This imbalance even remains 2 years after treatment with curative intent [6]. Consistently, a higher number of tumor infiltrating CD4+ and CD8+ lymphocytes is associated with better overall survival in HNSCC patients [7]. Additionally, natural killer cell (NK) function is impaired in HNSCC patients, which is accompanied by elevated levels of TGF beta and soluble major histocompatibility complex Class I chain-related peptide A [8, 9]. In human papilloma virus (HPV)-positive oropharyngeal carcinoma patients, an increased number of CD56+ cytotoxic NK cells was reported, which might contribute to the favorable prognosis of this subpopulation [10]. The role of regulatory FoxP3+ T‑cells (T regs) is still a matter of debate in HNSCC. A recent meta-analysis suggested that T regs are even favorable with respect to prognosis in HNSCC [11]. While the anti-inflammatory effect of T regs might contribute to this finding, only a subpopulation of T regs such as CD4+, CD25 high+, and FoxP3+, which were described to be elevated in HNSCC, are activated and act as potent immunosuppressors [12]. Finally, myeloid depressor cells (MDSC) and macrophages of the M2 phenotype are found in HNSCC tissues and peripheral blood facilitating an immunosuppressive state [5].

## Immune escape mechanisms in head and neck cancer

HNSCC cells apply certain strategies to escape immunosurveillance and subsequent elimination. On the one hand they interact indirectly with the immune system in order to maintain an immunosuppressive microenvironment. Secretion of cytokines such as TGF beta, interleukin 10, or VEGF establishes a tumor-promoting immunosuppressive environment [2]. Additional factors such as the secretion of interleukin 6, which prevents the activation of T cells, NK cells or dendritic cells maturation via STAT3, further modulates the cellular immune system resulting in conditions, which facilitate immune escape [2].

On the other hand a key component for immune escape of HNSCC cells is the reduction of their inherent immunogenicity by downregulating (but not complete loss) human leukocyte antigen (HLA) class I molecules and disrupting of the antigen-processing machinery (APM) [13]. Apart from that HNSCC exploit the fact that the immune system is tightly regulated through immune checkpoints in order to avoid autoimmunity or immune system over-activation under physiological circumstances.

## Immune checkpoints

**Fig. 2:**
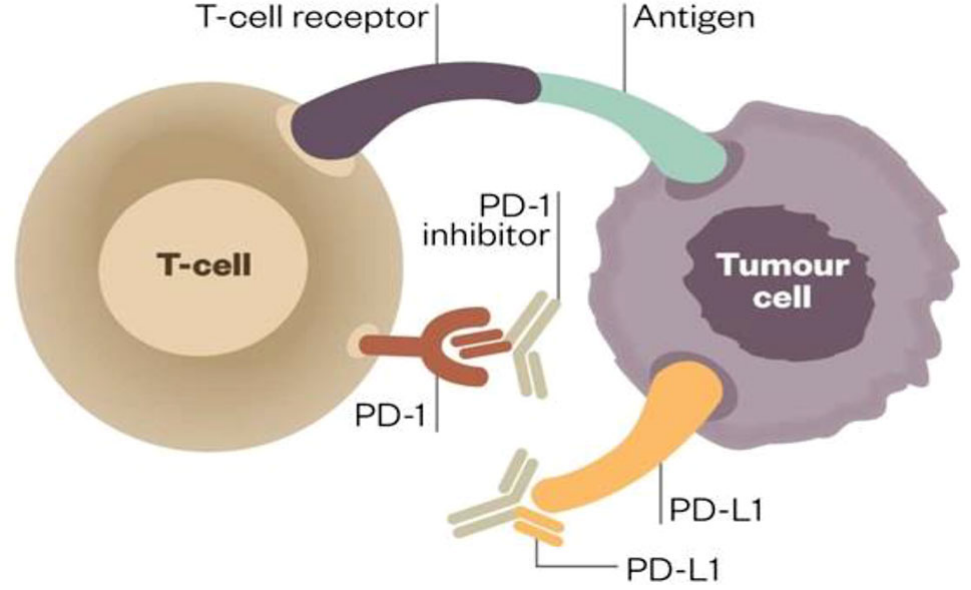
PDL-1/PD-1 interaction for T‑cell activity modulation

In recent years the introduction of immune checkpoint inhibitors for therapeutic purposes has revolutionized cancer treatment. T cell regulation, i. e., activation or inhibition is mediated via co-stimulatory or co-inhibitory signals. This interaction is exerted via ligand/receptor interaction. T cells harbor a myriad of both activating receptors such as OX-40, GITR, or CD 28 and inhibitory receptors (the so-called immune checkpoints) such as programmed death receptor 1 (PD-1) or cytotoxic T‑lymphocyte-associated protein 4 (CTLA-4) [14]. Activation of this immune checkpoints results in T cell deactivation (Fig. 2; [15]). Hijacking these pathways by tumor cells contributes to their successful immune escape. For HNSCC tumors it has been reported that 45–80 % express programmed death ligand 1 (PDL-1) [16]. In addition, exposure of HNSCC cells to therapy can result in tumor PDL-1 upregulation: It has been observed that HNSCC patients have elevated PDL-1 expression compared to healthy controls and that chemotherapy and radiation causes an PDL-1 upregulation in HNSCC patients, which lasts up to one year [17]. PDL-1 overexpression in tumor cells is induced via extrinsic interferon gamma secretion by NK or CD8+ cells or by intrinsic oncogenic drivers [18]. Very recently, the EGFR/JAK2/STAT-1 axis was identified for HNSCC to act as such a driver pathway [18]. Apart from that, HNSCC cells can respond to cetuximab treatment with recruitment of CD4+, FOXP3+ intra-tumoral T regs expressing CTLA-4, CD39, and TGFβ, which results in suppression of cetuximab-mediated antibody-dependent cellular cytotoxicity (ADCC) and poor prognosis [19]. Preclinical data indicate that CTLA-4 blockade with the monoclonal antibody ipilimumab might restore cetuximab sensitivity [20].

## Clinical immunotherapy data

Currently there are dozens of clinical trials evaluating the efficacy and safety of immune checkpoint inhibitors as monotherapy or in combination regimens in HNSCC. Table 1 lists selected clinical trials with PD‑1, PD-L1, and CTLA-4 inhibitors, which are tested in HNSCC at the moment. Data that the PD-1 antibody pembrolizumab might be effective in metastatic/recurrent (R/M) HNSCC patients were generated in the Keynote 12 phase I/II trial. This study had a head and neck cohort (inclusive expansion) and included 132 patients with R/M HNSCC, who were partially heavily pretreated (37 % had 3 or more lines of therapy). Patients received pembrolizumab 200 mg every 3 weeks. Primary objective was objective response rate (ORR), which was reported to be 24.8 %. Of note both HPV+ and HPV- patients responded to therapy [21]. Pembrolizumab therapy was very well tolerated. The majority of patients (59.8 %) had grade 1/2 adverse events (AE) with fatigue being the most predominant one. Severe immune-related AEs such as pneumonitis was reported in 5 patients (3.8 %) [21].

**Table 1:** Selected clinical immunotherapy trials in HNSCC patients

Clinical trial title	Phase	Target	Population
Pembrolizumab (MK-3475) Versus Standard Treatment for Recurrent or Metastatic Head and Neck Cancer (MK-3475-040/KEYNOTE-040)	III	PD-1	R/M HNSCC after platinum failure
A Study of Pembrolizumab (MK-3475) for First Line Treatment of Recurrent or Metastatic Squamous Cell Cancer of the Head and Neck (MK-3475 048/KEYNOTE-048)	III	PD-1	R/M HNSCC first line
Tolerance and Efficacy of Pembrolizumab or Cetuximab Combined With RT in Patients With Locally Advanced HNSCC (PembroRad)	II	PD-1 plus Radiation	Locally advanced HNSCC
Talimogene Laherparepvec With Pembrolizumab for Recurrent Metastatic Squamous Cell Carcinoma of the Head and Neck (MASTERKEY232)	I	PD-1 plus oncolytic virus	R/M HNSCC after platinum failure
Trial of Nivolumab vs Therapy of Investigator’s Choice in Recurrent or Metastatic Head and Neck Carcinoma (CheckMate 141)	III	PD-1	R/M HNSCC after platinum failure
Study of Nivolumab in Combination With Ipilimumab Compared to the Standard of Care (Extreme Study Regimen) as First Line Treatment in Patients With Recurrent or Metastatic Squamous Cell Carcinoma of the Head and Neck (CheckMate 651)	II	PD-1/CTLA-4	R/M HNSCC first line
Safety Study of Anti-LAG-3 With and Without Anti-PD-1 in the Treatment of Solid Tumors	I	PD-1/LAG-3	R/M HNSCC immunotherapy naive
Study of MEDI4736 Monotherapy and in Combination With Tremelimumab Versus Standard of Care Therapy in Patients With Head and Neck Cancer	III	PDL-1/CTLA-4	R/M HNSCC after platinum failure

Very recently the data of the randomized checkmate 141 phase III trial were presented [22]. This study investigated the efficacy of the monoclonal PD‑1 antibody nivolumab given every 2 weeks in platinum-refractory R/M HNSCC patients compared to investigator’s choice (cetuximab, methotrexate, or docetaxel). Primary objective was overall survival (OS). The study was stopped early due to superiority of the nivolumab arm. Patients receiving nivolumab had a median OS of 7.5 months and a 1-year OS rate of 36 % compared to 5.1 months and 16.6 % in the standard arm (HR 0.70; CI: 0.51–0.96; *p* = 0.0101) [22]. In the subgroup analysis it was shown that the survival benefit was more prominent in the p16+ group and especially patients with a PDL-1 expression >1 % benefited from the treatment. Of note the study was not designed to find OS differences with respect to p16 or PDL-1 status and was positive for the overall study population [22]. In terms of toxicity no new safety issues arose compared to the Keynote 012 trial.

Apart from checkpoint inhibitors several other immunotherapy strategies, which are not focus of this review article, such as adoptive cell transfer or cancer vaccines are in clinical development. Oncolytic viruses selectively target cancer cells, deliver antigens, and cause a host immune response. The attenuated herpes simplex virus talimogene laherparepvec (T‑VEC) has been recently approved for melanoma treatment and studies in HNSCC (in combination with checkpoint inhibitors) are underway [23].

## Biomarkers for checkpoint inhibitor therapy

Identification of predictive biomarkers in order to select patients, who benefit from checkpoint inhibitor treatment will be crucial. The first and obvious potential biomarker is the evaluation of PDL-1 expression in tumor tissue. However, several shortcomings are linked to this approach. First, there a technical issues, as there are several, partially not validated, PDL-1 antibodies available for immunohistochemistry and no cut-off level has been defined for positivity so far. Second, PDL-1 is a dynamic biomarker, which expression might vary even within the tumor tissue or depending on the time point. Finally, the observation that also patients, who do not show PDL-1 expression, might respond to checkpoint blockade is well documented [24]. In non-small cell lung cancer it has been shown that patients, who are heavy smokers, respond better to nivolumab compared to never smokers [25]. This phenomenon can be explained with a higher mutational burden in this patient population, which results in neo-antigen release and a subsequent boost in immunogenicity of the tumor. Since HNSCC cells were found to harbor one of the highest somatic mutation frequency among all solid tumors and similar molecular smoking patterns were identified in HNSCC patients, this concept might apply to HNSCC as well [26]. In addition, the role of PDL-2, another ligand of the PD-1 receptor is still poorly understood. Data from the keynote 12 trial showed that there is a discordance between PDL-1 and PDL-2 expression in a subpopulation of patients and suggest that PDL-2 expression is associated with higher ORR after adjusting for PDL-1 expression [27]. However, further research is necessary to incorporate these findings into the daily clinical practice.

## Conclusion and perspectives

No new drugs have been approved for HNSCC since the introduction of cetuximab more than 10 years ago. Phase III trial results of the checkpoint inhibitor trials are eagerly awaited and the approval of nivolumab based on the checkmate 141 data can be expected in 2016/2017. Trials evaluating combinations of checkpoint inhibitors with other costimulatory or inhibitory molecules or oncolytic viruses are ongoing. Finally, there is sound scientific rationale to incorporate immunotherapy in curative treatment protocols for non R/M HNSCC and to go for an immunotherapy combination with radio(chemo)therapy. All in all, immunotherapy offers exciting new perspectives for patients suffering from HNSCC after years of negative clinical trials.
